# ‘I Do It All Alone’: The Burdens and Benefits of Being Diagnosed With, and Treated for, Colorectal Cancer During the Covid‐19 Pandemic

**DOI:** 10.1111/hex.14110

**Published:** 2024-06-14

**Authors:** Christina M. Dobson, Jennifer Deane, Beth Osborne, Vera Araújo‐Soares, Colin J. Rees, Lorraine Angell, Linda Sharp

**Affiliations:** ^1^ Population Health Sciences Institute Newcastle University Newcastle‐upon‐Tyne UK; ^2^ Center for Preventive Medicine and Digital Health (CPD), Medical Faculty Mannheim Heidelberg University Mannheim Germany; ^3^ Independent Lay Researcher Newcastle upon Tyne UK

**Keywords:** cancer diagnosis, cancer pathways, cancer survival, colorectal cancer, Covid‐19, interviews, liminality

## Abstract

**Introduction:**

The Covid‐19 pandemic dramatically altered the way cancer care services were accessed and delivered, including for colorectal cancer (CRC). In the United Kingdom, patients were discouraged from presenting in primary care, many consultations took place remotely, investigative procedures and screening programmes were temporarily suspended, and fewer operations and treatments were delivered. People had to face the practical consequences of having cancer during a pandemic and navigate never before seen pathways, often alone. We examined the experience of being diagnosed and treated for CRC during the pandemic, and the implications of this on people's cancer journeys.

**Methods:**

Semi‐structured interviews were undertaken with people diagnosed with CRC during the Covid‐19 pandemic (January 2020–May 2021), in the North East of England. An iterative topic guide was used during interviews, which took place remotely (telephone or Zoom), were audio recorded, pseudo‐anonymised and transcribed. Initial transcripts were independently coded by two researchers, and a code ‘bank’ developed for application across transcripts. Development of themes and overarching analytical constructs was undertaken collaboratively by the research team.

**Results:**

Interviews were conducted with 19 participants, analysed and four key themes identified: (1) *The relative threats of Covid‐19 and Cancer* were not comparable, with cancer seen as posing a far greater risk than Covid‐19; (2) *Remote consultations* were problematic, affecting patients' abilities to build rapport and trust with clinicians, assess nonverbal communication, and feel able to disclose, comprehend and retain information; (3) *Stoma follow‐up care* was seen to be lacking, with long wait times for stoma reversal experienced by some; Finally, (4) *Being alone* during consultations negatively impacted some peoples' abilities to absorb information, and left them without the support of loved ones at an emotionally vulnerable time. However, some participants preferred being alone at certain points in their pathways, including receiving a diagnosis, and most frequently when receiving in‐patient treatment.

**Conclusion:**

Being alone brought unexpected benefits, absolving people from undertaking emotions work for others, and instead focus on their recovery, however, remote consultations negatively impacted patients' experiences. This study highlights the complex benefits and burdens of pandemic‐located cancer journeys, including how these shifted at different points across cancer pathways.

**Patient or Public Contribution:**

Lorraine Angell, a cancer survivor, has been central to this study from idea conception, contributing to: development of study focus and design; securing funding; production of patient‐facing materials; development of interview topic guides; analysis and interpretation of data; and drafting of key findings and manuscripts.

## 1 Introduction

The Covid‐19 pandemic abruptly altered healthcare configuration and delivery, not least for cancer care services [1]. Concerns about the impact of Covid‐safe measures on cancer diagnosis, management, and outcomes were quickly raised, with fears of tens of thousands excess cancer deaths on the horizon [2]. For colorectal cancer (CRC), the third most common cancer, and second most common cause of cancer death globally [3], disruption was felt at all stages of cancer journeys.

### 1.1 CRC Diagnosis and Treatment in the United Kingdom During the Covid‐19 Pandemic

In the United Kingdom, General Practitioners (GPs) act as gatekeepers to secondary care; the overwhelming majority of those diagnosed with cancer present initially with symptoms to their GP in primary care, who may then refer them for further investigation. The route through which patients are diagnosed (i.e., urgent or routine referrals from primary care, emergency presentations, or cancer screening) is directly associated with CRC outcomes [4]. Routes to diagnosis changed dramatically due to pandemic service reconfigurations, and a 13%–16% increase in CRC deaths was projected, equating to 1500 excess CRC deaths [5].

People's health behaviours changed during the pandemic with consultation rates for possible symptoms of cancer halving during the first wave [6]. Some people avoided primary care so as not to overburden the National Health Service (NHS) [7] (in line with strong public health messaging to do so), but many, particularly older individuals, did so to minimise their risk of contracting Covid‐19 [8, 9, 10]. People also appraised symptoms potentially indicative of CRC—such as fatigue, change in bowel habit, or weight loss—as being relatively trivial, in the wider context of the pandemic [9].

Not only did people's engagement with primary care change, but so did the delivery of primary health care. Remote consultations were rapidly introduced, which largely took place via telephone, but sometimes included video calls or secure photo upload [11]. For people presenting with symptoms suspicious of CRC, faecal immunochemical testing (FIT) was implemented, to triage those at greatest need of an urgent investigation and minimise burden on endoscopy services. In Spring 2020 primary care consultations and urgent referrals for suspected cancer were down by 60% [12].

In April 2020 all but emergency endoscopic procedures were temporarily suspended [13, 14, 15, 16]. The Bowel Cancer Screening Programmes (BCSP) were also temporarily suspended. When reinstated, uptake was reduced, as people balanced the risks of not being screened with the risk of contracting Covid‐19 whilst attending for diagnostic tests. Those who did engage, and had positive screening results, underwent assessment with specialist screening practitioners remotely, which negatively impacted the numbers of people agreeing to proceed to colonoscopy [17].

Covid‐19 also impacted delivery of cancer treatment. This resulted in a 22% reduction in cases commencing treatment, including laparoscopic surgery [18].

These unprecedented changes in cancer care delivery had the potential to drastically impact cancer outcomes and created an environment with potential to significantly alter patient experiences. People being investigated, diagnosed, and treated for CRC, had to navigate a reconfigured and changing health care system, whilst also assessing and managing risk of Covid‐19, and adjusting to the emotional and social consequences of both diseases. The purpose of this study was to investigate the experience of being diagnosed with, and treated for, CRC during the Covid‐19 pandemic, identifying learning to inform health care delivery now, and in the event of similar future events.

## 2 Methods

### 2.1 Recruitment

People diagnosed with CRC between January 2020 and May 2021 were identified by research nurses at South Tyneside & Sunderland NHS Foundation Trust, which serves a diverse catchment area across two hospital sites, including communities characterised by high deprivation (12th most income deprived local authority in the United Kingdom) [19]. Research nurses approached a diverse sample (in relation to route to diagnosis (symptomatic/screening), stage at diagnosis, and point in the pandemic at which they were diagnosed) to ensure that the study included people whose diagnoses occurred during different pandemic ‘waves’ and measures. Eligible individuals (aged 18 and over and with the capacity to provide informed consent) were contacted by telephone to ascertain willingness to receive a study pack providing further information. Those who wished to take part in the study returned their completed consent form to the Trust (14% response rate), where research nurses assigned study ID numbers and sent copies of consent forms, and (separately) pseudo‐anonymised clinical information, to the university research team. The university researchers contacted participants to arrange a suitable time for interview.

### 2.2 Fieldwork

A topic guide was developed by the research team, including lay co‐investigator LA, and informed by *The Model of Pathways to Treatment* [20]. The topic guide was iterative, ensuring that key questions were consistently covered across interviews, whilst also allowing the researcher flexibility to explore novel lines of inquiry and incorporate them into subsequent interviews. Interviews explored topics including symptom onset and appraisal, help‐seeking decision‐making, initial contact with the healthcare system (i.e., via primary care or screening), investigations, diagnosis, and treatment experiences, all located within participants' personal and social contexts, and the broader Covid‐19 landscape.

Interviews were conducted remotely (via telephone or video‐conferencing (i.e., Zoom)) by one of three trained, female researchers (CD, JD, BO), none of whom had a pre‐existing relationship with any participants. Interviews lasted between 35 and 80 min and took place 5–18 months after participants' diagnoses (mean 12 months). This meant that participants had completed, or were nearing completion of, treatment, allowing discussion of experiences across the entire cancer diagnostic and treatment pathway. Interviews were audio‐recorded, pseudo‐anonymised and transcribed verbatim.

Participants were initially sampled consecutively, with later purposive sampling for patients diagnosed through emergency routes. Twenty‐three individuals expressed an interest in being interviewed, two of whom later withdrew, and two of whom it was not possible to contact. Recruitment (and interviewing) ceased when data repeatedly supported emerging themes, and a point of ‘accuracy’ was felt to have been reached within the data set [21].

### 2.3 Analysis

The first six transcripts were read and re‐read by two team members of, (CD and JD; both experienced in qualitative analysis), who independently coded them, adopting an in vivo, line‐by‐line coding approach. Initial codes were compared and discussed, aligning codes amalgamated, and others refined, or rejected, to create a single code ‘bank’. This code ‘bank’ was then applied to all transcripts by either CD or JD. NVivo software was used to organise the data. Coded data was read and re‐read, comparing within and between cases to identify common themes and explore deviant cases, aided by the practice of memo‐ing [21, 22]. The researchers met regularly to discuss developing themes and overarching analytical constructs were developed further within meetings of the wider research group.

This study was given a favourable opinion by the London—Hampstead NHS Research Ethics Committee (REC) and Health Research Authority (HRA) approval on 10 March 2021 (IRAS ID: 291153).

## 3 Results

Interviews were undertaken with 19 participants (*see* Table 1 *for characteristics)*. Four interconnected themes, identified as key during the analysis, are presented here. These were: The Relative Threats of Covid‐19 and Cancer; Remote Consultations and Communication; Stoma Follow‐Up Care; and Being Alone.

**Table 1:** Participant characteristics.

Participant number	Gender	Age bracket	Route to diagnosis	Cancer stage	Month of diagnosis
001	Female	55–64	Urgent cancer referral	IV	February 2020
002	Female	65–74	Urgent cancer referral	Unknown	April 2020
003	Male	65–74	Urgent cancer referral	I	February 2021
004	Male	75+	Urgent cancer referral	III	February 2020
005	Female	55–64	Emergency admission	IV	March 2020
006	Female	65–74	Bowel Cancer Screening	I	September 2020
007	Male	55–64	Urgent cancer referral	III	November 2020
008	Female	75+	Bowel cancer screening	IV	April 2021
009	Female	65–74	Bowel cancer screening	II	November 2020
010	Female	65–74	Bowel cancer screening	I	October 2020
011	Female	65–74	Urgent cancer referral	III	September 2020
012	Male	65–74	Urgent cancer referral	I	September 2020
013	Male	65–74	Bowel cancer screening	II	September 2020
016	Male	75+	Emergency admission	II	August 2020
017	Male	55–64	Bowel cancer screening	I	April 2021
018	Male	65–74	Urgent cancer referral	I	April 2021
019	Male	75+	Bowel cancer screening	II	May 2021
020	Male	75+	Emergency admission	III	August 2020
021	Male	65–74	Urgent cancer referral	Unknown	October 2020

### 3.1 The Relative Threats of Covid‐19 and Cancer

Most participants did not see Covid‐19 as a pressing concern or threat, as worries about their cancer diagnosis far eclipsed worries about contracting Covid‐19.

*“*I'd just been diagnosed with a blockage, which I'd just been told was cancer of the bowel. So, Covid is the last thing on your mind, to be honest with you, when you get told something like that.*”*

(P007, 55–64‐year‐old male, diagnosed November 2020)

*“*I'm not going to die of Covid, because I'm going to die of cancer, so Covid is the least of my worries.*”*

(P001, 55–64‐year‐old female, diagnosed February 2020)

Most participants discussed adhering to social distancing guidelines throughout their cancer journeys and felt satisfied that these general precautions were adequate to minimise their risk of contracting Covid‐19. However, some participants felt that they would be more vulnerable to Covid‐19, should they contract it, on account of their cancer. For these people having to attend hospital in their capacity as a cancer patient was concerning: attending health care settings required them to trust not only the measures that had been implemented, but also trust that hospital staff and other patients had followed them appropriately.

*“*You're going in [to hospital] thinking ‘who else is in the hospital and who's treating me, who's the other patients in the waiting room, who's?’ you're just thinking what you're coming up against when you've been told to stay away from everybody and then all of a sudden you're in the hospital with hundreds of people milling about.*”*

(P012, 65–74‐year‐old male, diagnosed September 2020)

*“*You're going to the hospital, like in the waiting room, you know, you'd hope that everyone was sticking to all the rules and everything.*”*

(P008, 75+‐year‐old female, diagnosed April 2021)

Many participants reflected on media reporting of delays to cancer care; they spoke of this as a source of worry but, at the same time, did not feel they experienced these delays within their own diagnostic and treatment pathways.

*“*I see things in the news or I read things that Covid's having an impact on treatment and cancer treatments but it didn't for, I, I can't understand where it came from, or where that news comes from. It never had an impact on my treatment whatsoever.*”*

(P007, 55–64‐year‐old male, diagnosed November 2020)

*“*I hear these things on the television that people are waiting and not getting treatment and it was really scary listening to that, but I feel like my treatment was moving on.*”*

(P008, 75+‐year‐old female, diagnosed April 2021)

### 3.2 Remote Consultations and Communication

Participants frequently discussed their experiences of remote consultations, and many felt that remote consultations were inadequate. This was particularly true for primary care, where it was felt that GPs could not assess someone properly without a face‐to‐face appointment and physical examination.

*“*You just need to see a doctor face‐to‐face, he can judge what your problems are as well it gives him a better chance of examining you, and listening to you, and your facials….To me, the phone calls, they must miss a lot of cases. And some people say ‘oh well, he said that I'm alright, so I'm alright’ and they don't follow it forward and they carry on as they are and, really, that's the end of them for the majority.*”*

(P009, 65–74‐year‐old female, diagnosed November 2020)

Many people lacked confidence that the GP had assessed them accurately and managed them appropriately after telephone consultations. A small number of participants felt that remote primary care consultations had detrimentally impacted the timeliness of their diagnosis.

*“*If I'd gone to the doctors…instead of having a phone appointment, and actually gone to the GP to have a physical examination, then I would have probably been in the hospital, what, 4‐5 months earlier. So, basically, between April and September, that time had been lost really. I had another 4‐5 months sort of having cancer and it was obviously getting worse and worse, because it doesn't get any better does it.*”*

(P012, 65–74‐year‐old male, diagnosed September 2020)

In secondary care, participants described how remote consultations negatively impacted their ability to receive information; this was particularly an issue amongst people who had hearing difficulties.

*“*I do have hearing problems, like, you know, hearing aids in either side of my ears and it can be distressful that I don't catch every single word.*”*

(P004, 75+ male, diagnosed February 2020)

*“*I think it's perhaps more difficult for me because I'm deaf…normally do a lot of lip reading anyway, and like to watch people's facial reactions.*”*

(P005, 55–64‐year‐old female, diagnosed March 2020)

Irrespective of hearing impairment, all participants preferred in‐person hospital appointments as these enabled them to develop rapport and build a relationship with clinicians. In‐person appointments also allowed them to assess non‐verbal communication, such as body language and facial expressions, which made many more confident and comfortable to ask questions.

*“*I'd rather see him face‐to‐face…I think it's better, seeing him is more personal, seeing a doctor or a nurse face‐to‐face and you can see their expressions, what they're thinking.*”*

(P010, 65–74‐year‐old female, diagnosed October 2020)

*“*I didn't actually meet the oncologist for a whole year, it was all over the telephone…it's not a personal conversation…I think face‐to‐face ya have more of a, you get feedback, so then you ask more questions, and the feedback you get, you ask more questions again, but I think over the phone, it's just the basics.*”*

(P005, 55–64‐year‐old female, diagnosed March 2020)

Remote consultations did not just affect diagnostic and treatment pathways, but also shaped experiences of follow‐up care, with similar concerns raised about the adequacy of assessment.

*“*You are waiting 3 months to see a consultant and they put you over the phone, you don't know what's going on. You don't know if your wound is healing, you know, it's up to you to check it, but it would have been easier seeing a doctor.*”*

(P010, 65–74‐year‐old female, diagnosed October 2020)

### 3.3 Stoma Follow‐Up Care

Whilst most participants didn't feel that the pandemic had caused delays in their diagnostic and treatment pathways, this was different as regards follow up: participants described long waiting times for stoma care support and stoma reversal.

*“*I phoned my GP and they're saying the stoma nurses should be doing more; I phone the stoma nurse and they said they can't do anymore. So, you're sort of, at the moment, I've got an ongoing issue and I'm sort of being left to me own devices.*”*

(P017, 55–64‐year‐old male, diagnosed April 2021)

*“*I've had a stoma bag for 10 months and just waiting to hear if we get a phone call from the hospital to say that, erm, we're going to reverse the operation and reconnect the small bowel…we're just waiting now to get this stoma bag removed and then get on, hopefully, get on with normal life…*”*

(P012, 65–74‐year‐old male, diagnosed September 2020)

Whilst participants were cognisant of the fact that these procedures were not clinically urgent, some desperately wanted them, to allow them to return to a sense of normality.

### 3.4 Being Alone

One consequence of Covid‐19 safety measures was that most people had to attend hospital appointments alone, across the cancer diagnostic and treatment pathway. This experience was discussed in almost all interviews. Many participants received their diagnosis alone, without the opportunity to have a family member or friend present, for emotional or practical support.

*“*When you walk into the room and there are 3 nurses there, one was a Macmillan nurse…you knew it was bad news, you know what I mean. It was just horrendous that I had to do it by myself…I wanted to cry but I didn't cry because I wouldn't have stopped…. And all you can see is the masks on people's faces and just the eyes…. I think that was a scary part as well. Just doing it on my own….You can't take everything in. But, I thought if somebody was by your side, they'll take in and they ask you a question.*”*

(P010, 65–74‐year‐old female, diagnosed October 2020)

*“* **Were you alone when you received your diagnosis?**

Yes

**How did this make you feel?**

Decimated.*”*

(P004, 75+ male, diagnosed February 2020)

The diagnostic moment was pivotal and the absence of emotional support was felt acutely. The pandemic removed much opportunity to connect through non‐verbal communication, such as the ability to see someone's face because of masks, or the importance of human touch.

*“*She said ‘we're not supposed to touch with the COVID going on, but’ she said ‘I've got me gloves and that’ but she said, with me being in me seventies ‘your family's not around, I've taken time to hold your hand. You've got a bit of bad news’ but she said ‘I'm with you’. And it sounds stupid but that reassurance off that nurse…I thanked her very much.*”*

(P002, 65–74‐year‐old female, diagnosed April 2020)

For participants who managed to have someone attend appointments alongside them, these individuals provided not only emotional support, but, more importantly, aided with information assimilation.

*“*Me husband did come with me. I know a lot of the time he wasn't meant

to but, yes, he did come with me.

**Was this helpful that you had someone with you?**

Very much so, yes, because when you're in shock, you're not really taking

everything in that they're saying.*”*

(P005, 55–64‐year‐old female, diagnosed March 2020)

*“*I think it's invaluable to have somebody else with you because I don't think you take everything in, I don't anyway. My daughter told me things later that they said, or my son, and they said I hadn't sort of taken that in.*”*

(P008, 75+‐year‐old female, diagnosed April 2021)

Not all participants viewed being alone during their cancer diagnosis negatively, however. A small number considered that being alone absolved them from an unspoken obligation to manage others' emotional needs. One participant actively chose to take advantage of the opportunity Covid‐19 presented to attend such appointments alone.

*“*I preferred it [being alone] because erm, I knew the worst, like when I went to speak to the nurses to get the diagnosis, I wanted to do it myself, rather than the wife being there, because I knew she would break down. Erm, I mean she was in the car park and she wanted to come in but I said ‘no’ and the nurses said that, erm, ‘you should have someone with you’ and I said ‘no, I'd rather not, the wife is very emotional to start with’.*”*

(P017, 55–64‐year‐old male, diagnosed April 2021)

Isolation continued after the diagnosis, with many participants discussing how hospital restrictions prevented family and friends from visiting when they were an in‐patient waiting for, or recovering from, cancer treatment. For some people, the experience of having to go down for surgery, or endure recovery, alone was ‘scary’ and ‘lonely’ (P010, 65‐74‐year‐old female, diagnosed October 2020).

*“*When you've been diagnosed with something and know you're going in for an operation it's only natural that you're going to want friends and family with you and of course you can't have them.*”*

(P006, 65–74‐year‐old female, diagnosed September 2020)

For some people, separation from family and friends during recovery was distressing, as it made it harder to reassure and support worried family members. Being diagnosed following an emergency admission also meant that people had to tell loved ones their diagnosis over the phone.

*“*Not being able to have visitors while you're in hospital, just being able to talk to family over the phone, I mean they can't see how you are or anything, you're just sat there saying ‘yes, I'm fine, I'm fine, don't worry’. But they're worrying themselves sick because they can't come to visit you on the ward because of Covid.*”*

(P012, 65–74‐year‐old male, diagnosed September 2020)

*“*It's bad enough having to tell her [his wife], say face‐to‐face, but just [to tell her the diagnosis] on the telephone, it wasn't very good at all…I was alone in hospital for 8 and a half weeks….couldn't even see the wife.*”*

(P020, 75+‐year‐old male, diagnosed August 2020)

However, others welcomed this enforced isolation, as it enabled them to rest and recover, freeing them from the need to socialise with, or emotionally support, others.

*“*Actually, sometimes it was good because if you couldn't be bothered to talk to anyone you would just switch them [the phone] off, so it did have a bonus, it did have a good part. You know, when people come and sit by your bed all the time and you just get sick of people sometimes, that sounds awful doesn't it? But, you know, sometimes you just want to be left alone, don't you.*”*

(P006, 65–74‐year‐old female, diagnosed September 2020)

*“*There was no visitors and that was, possibly a, for me, a positive. Because it had been a, I'd had a sort‐of 14‐hour operation and to be honest it wasn't pleasant and I wouldn't really have wanted to see anybody the way I, I, was feeling, and the way I was.*”*

(P017, 55–64‐year‐old male, diagnosed April 2021)

Not only did participants see the absence of visitors as beneficial to themselves, but also for others; it meant that they weren't impacting on the lives and time of family and friends, who may have felt obligated to visit. It was also seen as beneficial to the nursing staff, as it gave them more time to care for patients.

*“*I do it all alone. I'm going to chemo alone. I do everything alone. I don't want to impact anybody's life.*”*

(P001, 55–64‐year‐old female, diagnosed February 2020)

*“*Nurses had plenty of time to be attentive…so they were 100% focused on patients…there was no sort of interruptions at all.*”*

(P017, 55–64‐year‐old make, diagnosed April 2021)

Outside of the hospital setting, participants spoke about not being allowed to have visitors in their homes, because of national Covid‐19 restrictions. They mourned the loss of in‐person emotional support that may otherwise have been provided by family and friends. Practical support that could be offered by friends and family was reduced, meaning that additional burden often fell on the patient's spouse.

*“*Me friends and me family couldn't really come and see me, so obviously that was an impact, whereas normally, when you're not very well, people come round to see how you are….the outer family weren't allowed to come and visit at all so, obviously [husband] he was doing a lot of the, he was the one going shopping and if we needed anything going for it, because I was barely leaving the house.*”*

(P005, 55–64‐year‐old female, diagnosed March 2020)

*“*Living on your own, I was quite isolated you know. My daughter came round, you know, as much as she could, but that would be for a quick cup of tea in the garden and in the end I was sitting in the conservatory and she was sitting in the garden with big puffer jackets on, you know, so you can't stay long sitting like that in the cold so, you know, when you are on your own it's quite tough really.*”*

(P008, 75+‐year‐old female, diagnosed April 2021)

## 4 Discussion

This study examined the experiences of people diagnosed with CRC during the Covid‐19 pandemic, exploring the impact of reconfigured health care services and pandemic safety measures on cancer journeys. People viewed Covid‐19 as less of an immediate threat than their cancer, although many were still wary of contracting it and sought to minimise risk by adhering to Covid‐19 safety guidance. Two key, novel findings from the analysis were the impacts of isolation and remote consultation on pandemic‐located cancer journeys.

### 4.1 Being Alone and Liminal

A novel and striking finding of this research was the complex and dynamic nature of how isolation impacted participants' experiences across their cancer journeys. Many participants found it difficult not having a family member or friend present in consultations, as it impacted their ability to comprehend and retain information about their disease and treatment. A study in the United States also reported that people diagnosed during the pandemic found attending appointments alone difficult, and HCPs had to make greater effort to ensure patients heard and retained information [23].

The period in which people are investigated for possible cancer is characterised by uncertainty and liminality [24], wherein they are simultaneously someone without cancer, and someone who may have cancer within them. A cancer diagnosis is not just a clinical category, but a social process [25], whereby the diagnosis and label are created collectively, through numerous interactions between patients, clinicians, friends, and family members. However, during the pandemic, many had to navigate this diagnostic process alone, without the social support and resources of others to help them comprehend information provided. When people are given a cancer diagnosis they transition from this pre‐diagnosis liminality, into a new realm of patienthood [26], a time at which their sense of self may be disrupted [27], as their mortality is brought into question. At this point, concerns and uncertainties about meaning, identity and the future abound, making it difficult to retain information, or actively engage in decision‐making processes. We argue that for those diagnosed during the pandemic ‘pre‐diagnosis liminality’ was further heightened, as they had to navigate the diagnostic moment and process alone.

After a CRC diagnosis many people spend time as an in‐patient receiving treatment; it was in this period that participants' experiences of, and responses to, being alone were notably different to their pre‐diagnosis experiences. Other research with cancer and heart failure patients in the United States and Ireland has shown that enforced isolation during the pandemic was not welcomed by patients. The lack of visitors was reported to be lonely, demotivating [28] and frightening [29]. A striking and novel finding from this study, however, was that some patients welcomed the respite that the ward‐visitation restrictions provided. These restrictions absolved them from an obligation to reassure and comfort family and friends, thereby releasing them from emotion work and emotional labour [30]. This meant that they did not have to abandon their own emotional needs to tend to those of their spouses/others [31], and instead were able to focus on their recovery.

Follow‐up stoma care was also found to be limited. As a result, people were thrust into a further period of liminality, in which treatment had ended, but they were still awaiting stoma reversal or support. These individuals were no longer in a clearly defined status of cancer ‘patient’ but were unable to fully transition to the identity of ‘survivor’ [32], whilst they awaited intervention that would physically and symbolically facilitate this transition. Being alone was experienced negatively most acutely during the liminal periods of participants' journeys, whereas isolation during the treatment phase, a time in which identity and role are clearer, actually brought some unexpected benefits.

### 4.2 Remote Consultations

Although a recent review [33] found that primary care telephone consultations are as effective as in‐person consultations, our participants lacked confidence in their GP's ability to effectively appraise them remotely. Participants reported that remote consultations during the investigative and diagnostic period affected information disclosure and receipt, particularly as people couldn't lip‐read or interpret non‐verbal communication. This made it more difficult to build rapport with health care providers, which de‐personalised care and reduced confidence and willingness to ask questions during consultations.

Our findings support an emerging body of literature evidencing how patients refrain from asking questions during telephone consultations [29, 34]. This is possibly because telephone calls take a formal, ‘business‐like’ format, and are expected to be much shorter [35]. In contrast, face to face appointments leave space for informal ‘chat’, which aids rapport building between patient and clinician. Remote consultations have been shown to be effective when an initial in–person consultation has already taken place, or when they happen via video consultation [36, 37]. Banbury et. al. found that patients who had video consultations were significantly more likely to feel that their appointment was just as effective as an in‐person consultation, than those who had telephone consultations [38], echoing the importance of non‐verbal communication and rapport in patient experience.

This study also challenges previous work that has shown that CRC patients have been satisfied with telephone follow‐up appointments in *secondary care* both pre [39] and post‐pandemic [40]. Our participants, by contrast, reported that they did not feel that clinicians were able to adequately assess their health and healing.

### 4.3 Strengths and Limitations

This study was delivered by an experienced, multidisciplinary team of researchers and clinicians, bringing diverse perspectives to data analysis and interpretation. Multiple researchers undertook the interviews (CD, JD, BO) and coding (CD, JD). Development of analytical themes and constructs was undertaken by the wider team, including lay co‐investigator LA, to ensure patient voice remained central.

Participants were recruited from a single NHS Trust, encompassing two hospital sites, serving an area characterised by high levels of deprivation, and a predominantly white population. As such, findings may not be generalisable across all settings. However, aspects of our findings align with research in other populations.

Interviews were conducted remotely, either by telephone or video conferencing. Just as participants reported finding it difficult to build rapport with clinicians during remote consultations, it may also be that some found it difficult to build rapport with the interviewer in this setting. Although some interviews were shorter, and felt slightly more formal, than the interviewers' experiences of in‐person interviews pre‐pandemic, the majority were in‐depth, had a natural rhythm and flow, and apparent rapport between the two parties. Participants shared considered reflections, and long and personal narratives, and the richness of data shared suggests that rapport was unlikely to have been badly compromised as a result of the mode of data collection.

## 5 Conclusion

This study described the experiences of people diagnosed with, and treated for, CRC during the Covid‐19 pandemic. Most participants did not feel that the pandemic detrimentally impacted their cancer pathways, and Covid‐19 was viewed as a lesser threat than participants’ cancers.

Remote consultations were generally unwelcomed, with participants raising concerns that both primary and secondary care appointments could not be effective, nor could clinical assessment be robust, without doctors seeing the patient. During diagnostic work‐up in secondary care, remote consultations were problematic as they altered the dynamic of the interaction, making it very difficult to receive, and reflect on information, and ask appropriate questions within the limited time available. With increasing adoption of remote consultations, it is vital we consider their impact on patient experience and care, and consider supplementary, or alternative modes to ensure that patients are adequately supported to disclose and obtain information, such as potential for follow‐up telephone calls, and awareness of the importance of non‐verbal communication during consultations.

Enforced isolation prevented participants from accessing social support from family and friends pre‐diagnosis. Future strategies, such as allowing people to ‘dial in’ to consultations that they cannot physically attend alongside the patient, may be beneficial. However, enforced isolation also absolved people from providing social and emotional support to others during the treatment phase. The impact of minimising ward visits on patients' recovery and wellbeing is an area which is worthy of further examination.

This work highlights the complexity of benefits and burdens brought by the presence of friends and family members across people's cancer journeys, and how these shift as people transition between liminal and fixed states.

## Author Contributions

**Christina M. Dobson:** conceptualisation, investigation, funding acquisition, writing–original draft, methodology, writing–review and editing, project administration, formal analysis. **Jennifer Deane:** investigation, writing–review and editing, formal analysis. **Beth Osborne:** investigation, writing–review and editing. **Vera Araújo‐Soares:** conceptualisation, writing–review and editing, funding acquisition. **Colin J. Rees:** conceptualisation, funding acquisition, writing–review and editing. **Lorraine Angell:** conceptualisation, investigation, formal analysis, writing–review and editing, funding acquisition. **Linda Sharp:** conceptualisation, funding acquisition, writing–review and editing.

## Conflicts of Interest

The authors declare no conflicts of interest.

## Data Availability

Redacted, anonymised data are available on request from the corresponding author. The data are not publicly available due to ethical restrictions.
